# Which Came First, the Head or the Brain?

**DOI:** 10.1371/journal.pbio.1001484

**Published:** 2013-02-19

**Authors:** Robin Mejia

**Affiliations:** Freelance Science Writer, Albany, California, United States of America

**Figure:**
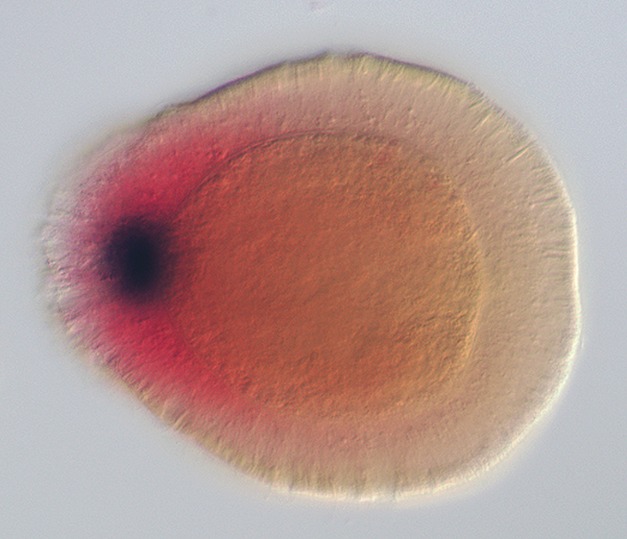
The “head development gene” *NvSix3/6* (red) and the apical organ gene *NvFGFa2* (blue) label distinct territories of the aboral domain of a three-day-old starlet sea anemone. Image credit: Chiara Sinigaglia and Fabian Rentzsch.

The sea anemone, a cnidarian, has no brain. It does have a nervous system, and its body has a clear axis, with a mouth on one side and a basal disk on the other. However, there is no organized collection of neurons comparable to the kind of brain found in bilaterians, animals that have both a bilateral symmetry and a top and bottom. (Most animals except sponges, cnidarians, and a few other phyla are bilaterians.) So an interesting evolutionary question is, which came first, the head or the brain? Do animals such as sea anemones, which lack a brain, have something akin to a head?

In this issue of *PLOS Biology*, Chiara Sinigaglia and colleagues report that at least some developmental pathways seen in cnidarians share a common lineage with head and brain development in bilaterians. It might seem intuitive to expect to find genes involved in brain development around the mouth of the anemone, and previous work has suggested that the oral region in cnidarians corresponds to the head region of bilaterians. However, there has been debate over whether the oral or aboral pole of cnidarians is analogous to the anterior pole of bilaterians. At the start of its life cycle a sea anemone exists as a free swimming planula, which then attaches to a surface and becomes a sea anemone. That free-swimming phase contains an apical tuft, a sensory structure at the front of the swimming animal's body. The apical tuft is the part that attaches and becomes the aboral pole (the part distal from the mouth) of the adult anemone.

To test whether genetic expression in the aboral pole of cnidarians does in fact resemble the head patterning seen in bilaterians, the researchers analyzed gene expression in *Nematostella vectensis*, a sea anemone found in estuaries and bays. They focused on the *six3* and *FoxQ2* transcription factors, as these genes are known to regulate development of the anterior-posterior axis in bilaterian species. (*six3* knockout mice, for example, fail to develop a forebrain, and in humans, *six3* is known to regulate the development of forebrain and eyes.)

The *N. vectensis* genome contains one gene from the *six3/6* group and four *foxQ2* genes. Sinigaglia and colleagues found that *Nvsix3/6* and one of the *foxQ2* genes, *NvFoxQ2a*, were expressed predominantly on the aboral pole of the developing cnidarian but, after gastrulation, were excluded from a small spot in that region (*NvSix3/6* was also expressed in a small number of other cells of the planula that resembled neurons). Because of this, the authors call *NvSix3/6* and *NvFoQ2a* “ring genes”, and genes that are then expressed in that spot “spot genes.” The spot then develops into the apical tuft.

Through knockdown and rescue experiments, the researchers demonstrate that *NvSix3/6* is required for the development of the aboral region; without it, the expression of spot genes is reduced or eliminated and the apical tuft of the planula doesn't form. This suggests that development of the region distal from the cnidarian mouth appears to parallel the development of the bilaterian head.

This research demonstrates that at least a subset of the genes that cause head and brain formation in bilaterians are also differentially expressed in the aboral region of the sea urchin. The expression patterns are not identical to those in all bilaterians; however, the similarities suggest that the patterns of gene expression arose in an ancestor common to bilaterians and cnidarians, and that the process was then modified in bilaterians to produce a brain. So to answer the evolutionary question posed above, it seems that the developmental module that produces a head came first.

**Sinigaglia C, Busengdal H, Leclère L, Technau U, Rentzsch F (2013) The Bilaterian Head Patterning Gene** ***six3/6*** **controls Aboral Domain Development in a Cnidarian. doi:10.1371/journal.pbio.1001488**

