# Correction: Which Came First, the Head or the Brain?

**DOI:** 10.1371/annotation/4a8d9f38-1d0d-4389-a284-9f2564e1ac0b

**Published:** 2013-03-05

**Authors:** Robin Mejia

There was an error in the first sentence of the last paragraph of this synopsis. The correct sentence is: This research demonstrates that at least a subset of the genes that cause head and brain formation in bilaterians are also differentially expressed in the aboral region of the sea anemone. 

